# Scientific Status Quo of Small Renal Lesions: Diagnostic Assessment and Radiomics

**DOI:** 10.3390/jcm13020547

**Published:** 2024-01-18

**Authors:** Piero Trovato, Igino Simonetti, Alessio Morrone, Roberta Fusco, Sergio Venanzio Setola, Giuliana Giacobbe, Maria Chiara Brunese, Annarita Pecchi, Sonia Triggiani, Giuseppe Pellegrino, Giuseppe Petralia, Giacomo Sica, Antonella Petrillo, Vincenza Granata

**Affiliations:** 1 Radiology Division, Istituto Nazionale Tumori-IRCCS-Fondazione G. Pascale, 80131 Naples, Italy; piero.trovato@istitutotumori.na.it (P.T.); igino.simonetti@istitutotumori.na.it (I.S.); s.setola@istitutotumori.na.it (S.V.S.); a.petrillo@istitutotumori.na.it (A.P.); v.granata@istitutotumori.na.it (V.G.); 2 Division of Radiology, Università degli Studi della Campania Luigi Vanvitelli, 80138 Naples, Italy; alessio.morrone@unicampania.it; 3 Medical Oncology Division, Igea SpA, 80013 Naples, Italy; 4 Italian Society of Medical and Interventional Radiology (SIRM), SIRM Foundation, Via della Signora 2, 20122 Milan, Italy; 5 General and Emergency Radiology Department, “Antonio Cardarelli” Hospital, 80131 Naples, Italy; giuliana.giacobbe@unicampania.it; 6 Diagnostic Imaging Section, Department of Medical and Surgical Sciences & Neurosciences, University of Molise, 86100 Campobasso, Italy; mariachiarabrunese@gmail.com; 7 Department of Radiology, University of Modena and Reggio Emilia, 41121 Modena, Italy; annarita.pecchi@unimore.it; 8 Postgraduate School of Radiodiagnostics, University of Milan, 20122 Milan, Italy; sonia.triggiani@unimi.it (S.T.); giuseppe.pellegrino@unimi.it (G.P.); 9 Department of Medical Imaging and Radiation Sciences, IEO European Institute of Oncology IRCCS, Via Ripamonti 435, 20141 Milan, Italy; giuseppe.petralia@ieo.it; 10 Radiology Unit, Monaldi Hospital, Azienda Ospedaliera dei Colli, 80131 Naples, Italy; giacomo.sica@ospedalideicolli.it

**Keywords:** small renal masses, ultrasound, contrast-enhanced ultrasound, computed tomography, magnetic resonance imaging, Bosniak classification, radiomics

## Abstract

**Background:** Small renal masses (SRMs) are defined as contrast-enhanced renal lesions less than or equal to 4 cm in maximal diameter, which can be compatible with stage T1a renal cell carcinomas (RCCs). Currently, 50–61% of all renal tumors are found incidentally. **Methods**: The characteristics of the lesion influence the choice of the type of management, which include several methods SRM of management, including nephrectomy, partial nephrectomy, ablation, observation, and also stereotactic body radiotherapy. Typical imaging methods available for differentiating benign from malignant renal lesions include ultrasound (US), contrast-enhanced ultrasound (CEUS), computed tomography (CT), and magnetic resonance imaging (MRI). **Results:** Although ultrasound is the first imaging technique used to detect small renal lesions, it has several limitations. CT is the main and most widely used imaging technique for SRM characterization. The main advantages of MRI compared to CT are the better contrast resolution and tissue characterization, the use of functional imaging sequences, the possibility of performing the examination in patients allergic to iodine-containing contrast medium, and the absence of exposure to ionizing radiation. For a correct evaluation during imaging follow-up, it is necessary to use a reliable method for the assessment of renal lesions, represented by the Bosniak classification system. This classification was initially developed based on contrast-enhanced CT imaging findings, and the 2019 revision proposed the inclusion of MRI features; however, the latest classification has not yet received widespread validation. **Conclusions**: The use of radiomics in the evaluation of renal masses is an emerging and increasingly central field with several applications such as characterizing renal masses, distinguishing RCC subtypes, monitoring response to targeted therapeutic agents, and prognosis in a metastatic context.

## 1. Introduction

Small renal masses (SRMs) are referred to as contrast-enhanced kidney lesions with a maximum diameter less than or equal to 4 cm, which can usually be consistent with stage T1a renal cell carcinomas [1,2,3,4]. Over the last few years, the detection of small and asymptomatic renal lesions (and consequently also of cancers) has increased globally partly due to the increased use of cross-sectional imaging [1,5]. Currently, 50–61% of all renal tumors are incidentally found, in comparison with only 13% in the 1970s [6,7].

Renal masses can be distinguished into two broad categories: tumors and pseudotumors. The latter are lesions that consist in non-neoplastic tissues, although at imaging could simulate cancer lesions [8]. There are many examples of renal pseudotumors, which include developmental abnormalities (prominent Bertin’s column, persistent fetal lobulation, dromedary hump, splenorenal fusion, renal cross ectopia, and renal hilar labrum), infectious and inflammatory processes (renal abscess, pyelonephritis, scarred kidney, renal granulomatous disease, renal tuberculosis, xanthogranulomatous pyelonephritis, renal sarcoidosis, and renal malacoplakia), vascular processes (extramedullary hematopoiesis, renal arteriovenous malformation, and renal hematoma), and miscellaneous lesions (regenerating nodules after reflux and post partial nephrectomy) [9,10,11,12]. For a correct differential diagnosis between tumors and pseudotumors, it is critical to refer to the patient’s medical history: an accurate history allows, in most cases, to lean toward the inflammatory nature, or the vascular or post traumatic nature of the renal lesions [13,14].

Lesion features influence the choice of management, such as nephrectomy, partial nephrectomy, ablation, surveillance, and also stereotactic body radiotherapy [15,16,17,18,19]. In addition, it should be considered that up to 20% of SRMs are benign [15] and that the risk of malignancy rises with increasing size [20,21,22]. For these reasons, an adequate characterization of renal lesions through the different imaging techniques is critical to optimize SRM management and to improve patient outcomes, preserving renal function in the best viable way and avoiding the risk of overtreatment [23,24,25,26,27,28,29,30].

Typical imaging methods for differentiating benign from malignant renal masses include ultrasound (US), contrast-enhanced ultrasound (CEUS), computed tomography (CT), and magnetic resonance imaging (MRI) [31,32,33,34]. 

The purpose of this narrative review is to report the state-of-the-art methods of imaging in SRM evaluations, including the indications, diagnostic possibilities, limits, and advantages of the different methods and typical findings researched, as well as future, prospective studies.

## 2. Ultrasound Assessment

Usually, ultrasound is the initial imaging tool used to detect small (and usually incidental) renal lesions [35]. US is a widely accessible, inexpensive, and noninvasive method [36] and is a very sensitive technique for detecting renal masses, being generally reliable in differentiating between solid and cystic lesions [37,38]. In fact, simple and uncomplicated renal cysts have a typical US appearance: well-confined anechoic lesions with thin walls and without septa and vascularity; posterior acoustic shadowing may be present [39]. Sometimes, there may be a few thin septa (5% of cases) or lesser amounts of intracystic hemorrhage/debris (5% of cysts) [40]. On the other hand, when the cystic lesions have thickened and irregular walls or septa with or without vascularization are observed on color and spectral Doppler imaging, they are defined as complicated cysts; these findings are suggestive of renal cell carcinomas (RCCs) and require further evaluation [41]. Therefore, it is evident how conventional US has some limitations in evaluating complex cystic, especially if small [42,43]. Furthermore, B-mode US has significant limitations also in the evaluation of solid lesions (especially in cases of small masses), except for angiomyolipomas (AMLs), which generally appear as hyperechoic and homogenous cortical lesions with sharp margins and with posterior acoustic shadowing [42,43]. However, in several cases, AMLs may also be atypical, appearing hypoechoic or mildly hyperechoic [35]. In addition, it should be noted that conventional US is not always able to differentiate AMLs from RCCs. In fact, although RCCs typically appear as hypoechoic, in 32% of cases, RCCs are hyperechoic, homogeneous, and with defined margins, thus being indistinguishable from small ALMs [37].

Another critical lesion is oncocytoma, which is generally a well-circumscribed mass in which a central scar may sometimes be visible [44]. In US studies, oncocytomas show variable echogenicity: several studies report a hypoechogenic appearance in 46% of cases, hyperechogenic in 23% of cases, iso-hyperechogenic in 8% of cases, and iso-echogenic in 8% of cases [45]. According to other case reports, the most frequent appearance is iso-echogenic to normal renal parenchyma [44].

**Figure 1:**
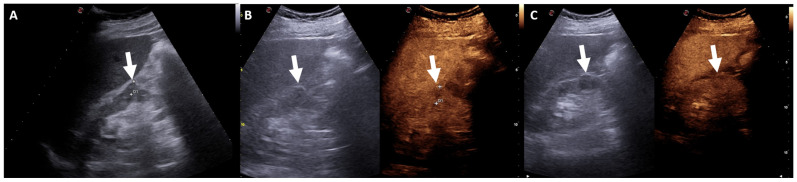
Renal ultrasound examination, B-mode (**A**) and CEUS scans (**B**,**C**). Small renal cortical lesion isoechoic to adjacent parenchyma (arrows). After an injection of intravenous contrast medium, the lesion shows enhancement.

On a color Doppler integration, RCCs, AMLs, and oncocytomas can show discrete hypervascularization [45]. These limitations can be decreased using contrast medium, which allows for better characterization of small renal lesions (Figure 1) [46].

## 3. Contrast-Enhanced Ultrasound Assessment

CEUS is a US technique characterized by the intravenous injection of contrast agents [47]. Second-generation US contrast agents include microbubbles of perfluorocarbon, nitrogen gas, or sulfur hexafluoride stabilized in a phospholipid membrane [48]. The microbubbles do not spread across vessel endothelium into interstitium and thus remain entirely intravascular [49]. These features allow for the optimal assessment of both the microcirculation and macrocirculation of the parenchyma and renal masses [50].

Several studies report increased diagnostic reliability in kidney cancers via CEUS, due to the improved visibility of renal lesions and effective delineation of tumor micro vessels [51]. In addition, it should be noted that microbubbles have an excellent safety profile as they have a lower incidence of collateral effects, such as nephrotoxicity; for these reasons, they may be utilized in patients with altered kidney functions [52]. Therefore, CEUS may also be especially useful in the diagnosis and assessment of SRMs, especially in differentiating between complex cysts, AMLs, and RCCs [52].

**Figure 2:**
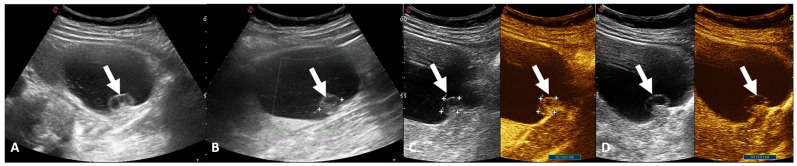
Renal ultrasound examination, B-mode (**A**,**B**) and CEUS (**C**,**D**) scans. Renal cystic lesion with small iso-hypoechoic wall nodule (arrows). After an intravenous contrast injection, the nodule shows enhancement. These features are suggestive of Bosniak Class IV.

In the assessment of complicated cysts, CEUS can be used to highlight the vascularization of septa and nodular protuberances (Figure 2), thus being able to help distinguish between benign cysts, indeterminate cysts, and cysts of obvious malignant appearance [47]. Regarding AMLs, these lesions, typically, show peripheral contrast enhancement and reduced central enhancement, compared with the renal cortex [47]. Instead, RCC typically presents heterogeneous hypervascularization in the arterial phase and early washout in the late phase; perilesional border enhancement is also one of the most frequent features of RCC [47]. Like RCCS, oncocytomas are hyper-enhancing lesions, but with delayed venous wash-out [45]. Hence, CEUS is a useful tool for lesion characterization.

## 4. Computed Tomography Assessment

CT is the main imaging method used to characterize SRMs, since it has an easy accessibility and high tolerability by patients [53,54,55,56]. However, since this technology is based on ionizing radiation, it should be chosen with caution for young patients and pregnant women and mostly used for lesion surveillance.

The CT evaluation of renal masses requires a multiphasic protocol [57,58,59]. However, it should be clarified that there is not a consensus on a specific protocol, so that for several departments, it is sufficient to include only a non-contrastographic and a nephrogenic phase, while for others, a corticomedullary and/or an excretory phase should be included [59].

The non-contrastographic phase is most useful for determining the presence, in a homogeneous mass, of macroscopic fat and/or calcifications [60]. With regard to the corticomedullary phase, several studies have shown that this contrast phase may be useful for differentiating subcategories of RCCs; in addition, it is most helpful for evaluating vessel anatomy, vascular tumor involvement, and arterial variants for surgical planning [61,62]. The nephrogenic phase provides the most accurate assessment of kidney parenchyma, as maximum and homogeneous enhancement are achieved, and for demonstrating an abnormal enhancement of renal masses [61]. The excretory phase is less useful for characterizing renal lesions; however, it plays a key role in assessing the anatomy of the calyces, renal pelvis, and ureters, especially in partial nephrectomy candidates [61].

The main CT findings of renal masses are attenuation (lesion density and detection of macroscopic fat), qualitative and quantitative enhancement, detection of central scars, and growth rate [15,60,63,64].

### 4.1. Lesion Density

The density of renal parenchyma is usually about 30 to 40 HU; in the non-contrastographic phase, the hyperattenuating lesion density ranges between 40 and 90 HU [65,66]. It has been reported that renal masses characterized by an unenhanced homogeneous density of more than 70 HU represent hemorrhagic cysts in more than 99% of cases [67].

### 4.2. Macroscopic Fat

Macroscopic fat is better seen on nonenhanced CT, where its density measures between −10 and −100 HU [68]. The finding of macroscopic fat within a solid renal mass is strongly suggestive of an AML, but not pathognomonic [69]. AMLs are the most frequent benign kidney tumors and are lesions containing muscular, adipose, and vascular tissue in varying proportions. They are usually rich in macroscopic fat, but, in 3–4.5% of cases, are atypical and contain microscopic fat not evident on CT [70,71].

### 4.3. Enhancement

Enhancement is the most important parameter that a radiologist should evaluate to characterize a renal lesion [72,73]. In fact, after the contrast administration, if a renal lesion has a density increase of more than 20 HU, this pattern is indicative of the presence of a solid component [74]. In cases where the increment is less than 10 HU, the lesion is classified as non-enhancing; when the increment is between 10 and 20 HU, the mass is defined as indeterminate, and further evaluation is required [75].

Generally, SRMs have homogeneous enhancement, whereas larger lesions undergo irregular enhancement due to necrotic components; the clear cell subtype typically shows more intense enhancement [76,77]. The most significant limitation of an enhancement assessment is the assessment of very small lesions, where it may be difficult to obtain a correct enhancement assessment as image artefacts may influence the results [76].

### 4.4. Central Scar

The central stellate scar is a typical but unreliable feature of oncocytomas [78]. In fact, according to several authors, less than half of oncocytomas have a central scar, while according to others, it is present in only 11% of cases [79,80]. Furthermore, necrotic components of RCC can mimic the scar. Therefore, no CT findings exist that can reliably distinguish an oncocytoma from an RCC.

### 4.5. Growth Rate

Growth rate assessment has limited validity in differentiating benign from malignant renal lesions [15]. In fact, small renal tumors have been shown to exhibit slow growth, regardless of histopathologic subtype, with reported average growth rates of 0.28 cm/year (range of 0.09–0.86 cm/year) [81]. Several studies report that 70% of SRMs undergoing surveillance via imaging methods have no measurable growth until 32 months [82,83]. Other authors have shown no statistically significant differences in growth rate between small RCCs and oncocytomas [81,84]. However, a rapid growth rate during the first year of surveillance via imaging methods may be suggestive of an aggressive tumor.

## 5. Cystic Renal Masses and Bosniak Classification

Renal cystic lesions are commonly detected in clinical practice, as they are estimated to occur in about 50 percent of people over the age of fifty [85]. However, it has been shown that 6% of asymptomatic renal cystic lesions are renal cystic neoplasms [86]. For these reasons, it is necessary to use a reliable method for the evaluation of renal cystic masses, represented by the Bosniak classification system [87].

Bosniak classification was reported for the first time in 1986 and has been widely accepted and utilized by both radiologists and urologists for the purpose of addressing the clinical problem of evaluating renal cysts [80,88,89,90,91,92,93,94]. It was subsequently updated in 2005 and 2019 [88,89].

It was initially developed based on contrast-enhanced CT imaging features, but the 2019 revision proposed the inclusion of MRI findings; however, the latest classification has not yet received widespread validation [90,91]. Some studies show greater sensitivity in CEUS compared with CT or MRI in evaluating intralesional septa [92,93].

Regarding the 2019 version, this method uses the classification of cystic lesions with the term “class” and not “category” and always uses Roman rather than Arabic numerals [89]. In addition, the 2019 Bosniak classification provides the definition of a “cystic renal mass” as a mass characterized by the presence of less than about 25% of enhancing components on subjective visual assessment [94].

**Figure 3:**
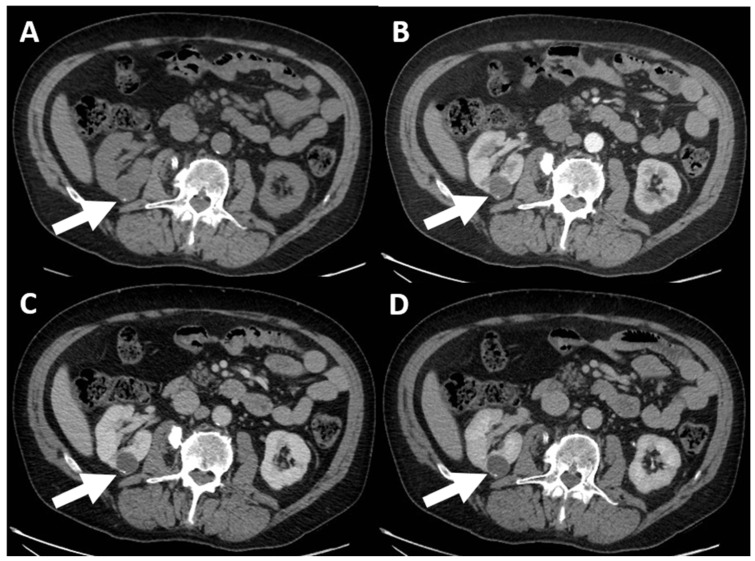
CT evaluation of small right renal cortical cystic lesion. The non-contrastographic (**A**), corticomedullary (**B**), nephrogenic (**C**), and excretory (**D**) phases show homogeneous cystic lesion with non-enhanced thin walls and calcification (arrows). The findings are consistent with Bosniak Class II.

According to the 2019 classification, cystic masses are divided into five classes:

- (a) Class I: benign simple cyst, which includes a mass with a well-defined, smooth and thin wall (≤2 mm), homogeneous and simple fluid content (−9 to 20 HU), without septa or calcifications, and with possible wall enhancement [89].
- (b) Class II: benign cyst, “minimally complex”, which includes 6 types in CT examination (Figure 3), all represented by well-defined masses with thin (≤2 mm) and smooth walls [95], and these include the following:
  1. Masses with thin walls (≤2 mm) and from one to three septa with possible enrichment of the septa and the wall, with the possible presence of calcifications of all types;
  2. Homogeneous masses with high density (≥70 HU) on non-contrast scan;
  3. Homogeneous masses with density >20 HU, which do not enhance and may have calcifications of all types;
  4. Homogeneous masses with density between −9 and 20 HU on non-contrast CT;
  5. Homogeneous masses with density between +21 and +30 HU at portal phase;
  6. Homogeneous masses with low density and too small to be characterized.

Bosniak I and II cystic masses do not need additional investigation or follow-up as they have a malignancy rate of about 0% [96].

**Figure 4:**
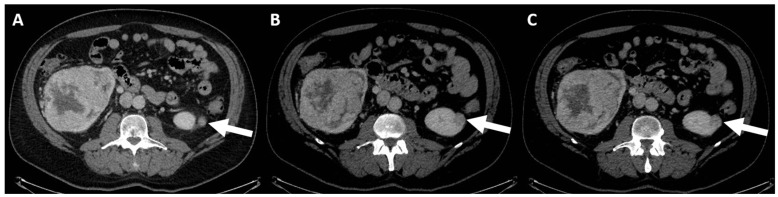
CT scan with intravenous contrast, corticomedullary phase (**A**–**C**): small left cortical enhancing renal lesion in patient with right renal tumor (arrows). The findings are consistent with Class III Bosniak.

**Figure 5:**
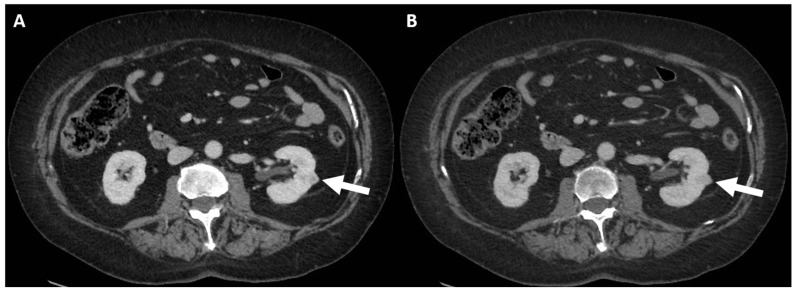
CT scan with administration of intravenous contrast, corticomedullary (**A**) and nephrogenic (**B**) phases: histologically confirmed renal cancer (arrows).

- (c) Class IIF: probably benign cyst masses that still require follow-up (F for follow-up) because they have a malignancy rate ranging from 5 to 17% [96,97]. This class comprises minimally complex cystic masses with mildly thickened (3 mm) and enhancing wall, or with mild and smooth thickening (3 mm) of one or more enhancing septa, or many (≥4) smooth and thin enhancing septa [89]. The necessary finding to define Class IIF or higher is the presence of measurable enhancement [91]. Follow-up is performed via US/CT/MRI methods, and there are no strict rules regarding timing: it is reasonable to do it at 6 months, at 12 months, and then annually for a total 5 years to assess any morphological changes [98].
- (d) Class III: indeterminate cystic mass, which includes cystic masses characterized by one or more thickened (≥4 mm) or enhancing and irregular (≤3 mm and with convex marginal protrusions) walls or septa [91]. Bosniak III masses (Figure 4) are “potentially” malignant in that they have an intermediate probability of malignancy (about 55%) [97]. Therefore, urologic consultation should be considered for possible partial nephrectomy or radiofrequency ablation in candidates unfit for surgery [99].

- (e) Class IV: clearly malignant cystic mass, which includes masses characterized by the presence of one or more enhancing nodules (≥4 mm convex protrusion with obtuse margins, or a convex protrusion of any size that has acute margins). A Bosniak IV mass (Figure 5) has a malignancy rate of about 90% and therefore requires urologic consultation to perform partial or total nephrectomy [100].

The 2019 version of Bosniak classifications also quantifies the number of septa: the term “few” is used in the case of 1–3 septa, while the term “many” is used in cases of four or more septa [101]. The thickness of the septa is also quantified: it is defined as “thin” in the case of thickness less than or equal to 2 mm, “minimally thickened” in the case of thickness equal to 3 mm, and it is defined as “thick” when equal to or greater than 4 mm [101]. A “wall or septal irregularity” is defined in the case of an enhancing convex protrusion with an obtuse margin less than or equal to 3 mm, while a “nodule” is defined as an enhancing convex protrusion with an acute margin of any size or an obtuse margin of 4 mm or more [101].

## 6. MRI Assessment

Although CT is the most-performed technique for the study of SRMs, MRI is considered by the American College of Radiology (ACR) to be not only a comparable and alternative technique to CT, but also a technique that can offer many advantages over CT [60,102]. The principal MRI advantages include (a) better contrast resolution and tissue characterization; (b) the use of functional assessment; (c) the possibility of performing the examination in patients who are allergic to iodinated contrast agents; and (d) the lack of ionizing radiation [103,104]. The latter aspect is critical in patients who frequently undergo imaging evaluations for RCC screening, such as in cases of Von Hippel–Lindau disease [105,106,107,108]. In patients with end-stage renal failure, moreover, unenhanced MRI presents more information regarding the evaluation of SRMs than unenhanced CT [109]. In addition, several studies have demonstrated the greater ability of MRI than CT in characterizing SRMs [102].

A study protocol includes T2-weighted (W) turbo spin echo (T2W) in three planes, chemical shift imaging (CSI), i.e., axial T1W in and out of phase (IP + OP), and fat-saturated 3D T1W gradient echo (FS) before and after gadolinium injection [110]. The combination of these sequences with dynamic contrast enhancement (DCE) and diffusion-weighted imaging (DWI) outlines a multi-parametric (MP) MRI protocol [111,112,113,114,115].

### 6.1. T2W Imaging

T2W sequences are typically performed as breath-hold (BH) half-Fourier single-shot turbo spin-echo (ss-TSE) in three orthogonal planes [116,117]. The use of ss-TSE results in improved spatial and contrast resolutions and reductions in artefacts and examination time [116].

T2W imaging is crucial in differentiating solid masses from cystic masses; it also may be useful in characterizing indeterminate solid masses [32]. In fact, a solid renal lesion characterized by heterogeneous hyperintensity with T2W compared with the renal cortex is more likely indicative of RCC, especially the clear cell subtype [118,119]. The evaluation of the signal intensity ratio of the lesion to renal parenchyma is highly dependable for differentiating RCC from AML [119].

**Figure 6:**
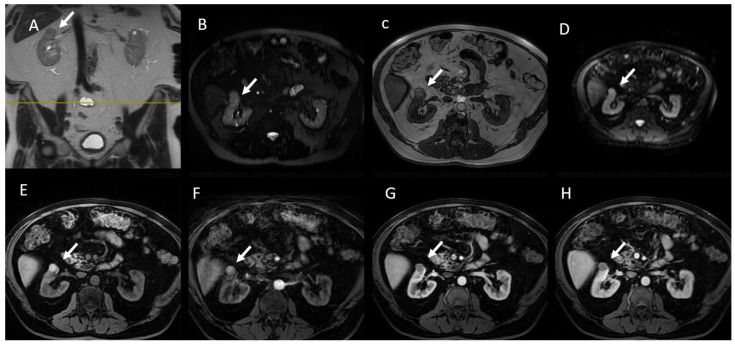
MRI assessment of small renal lesion: hemorrhagic cysts (arrows). The lesion is hypointense on T2W sequences (**A**,**B**) and hyperintense in T1W sequences without contrast agents (**C**,**E**), with restricted diffusion (**D**) and without contrast enhancement during contrast study ((**F**): arterial phase; (**G**): corticomedullary phase and (**H**): portal phase).

AML typically presents a low T2W signal intensity (SI) [120]. However, it should be remembered that RCC (especially the papillary subtype) and hemorrhagic cysts (Figure 6) may also be hypointense in T2; in fact, histologic confirmation is sometimes required to differentiate AML from RCC [121,122,123]. Instead, the differential diagnosis between solid neoplasms and hemorrhagic cysts is easier as the latter shows a high T1W signal and no enhancement [124].

### 6.2. CS (IP D OP) Imaging

It is well known that around 80% of clear cell RCC have a decreased SI in CS due to the presence of intracellular or intracytoplasmic lipids [125,126,127,128,129]. A significant decrease in SI index (SI CSI index > 25%) in RCC is typical of the cc-RCC subtype, whereas only rarely is there a minimal decrease in the remaining RCC subtypes [130]. On the other hand, evidence of gross/macroscopic fat on FS MRI sequences is considered diagnostic of AML [131,132,133]. However, although rarely, RCCs may show extracellular fat, due to varied reasons, including bone metaplasia and renal sinus fat engorgement [134]. Similarly, about 5 percent of AMLs do not contain an adequate amount of fat (minimally fat or fat-poor AMLs). Therefore, the evidence of signal loss in CS-MRI is not specific, as it can be detected in both RCC and AML [135,136,137,138,139,140,141,142,143,144,145,146]. In these cases, the assessment of different sequences can help in their characterization: the combination of the CS signal drop and evidence of macroscopic fat in the mass is indicative of classic AML [135,136], while the evidence of signal drop in a heterogeneously hyperintense mass in T2W, with contrast enhancement during the contrast study is pathognomonic for cc-RCC [137,138].

### 6.3. Diffusion-Weighted Imaging

Diffusion-weighted imaging, as it is known, utilizes the random movement of water molecules in several tissues to assess tissue cellularity [139]. An FS single-shot spin-echo echo-planar imaging (EPI) sequence is used [140,141].

**Figure 7:**
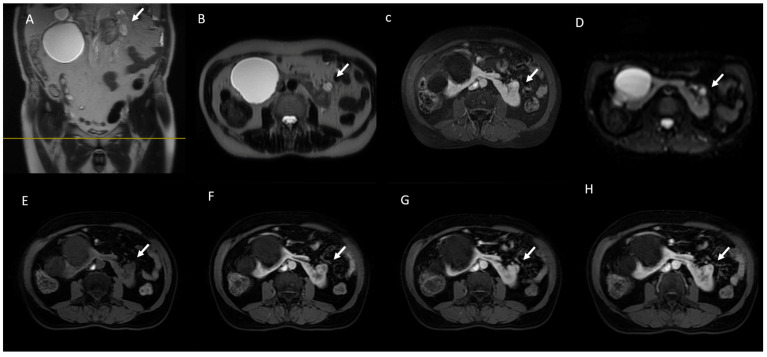
MRI assessment of low-grade clear cell RCC (arrows). (**A**) Coronal and (**B**) axial T2-W images show a small renal mass with a hyperintense signal, without FS in T2-W FS (**C**) and restricted diffusion (**D**). (**E**) Axial FS gradient T1W image shows hypointense lesion that after using a contrast agent, has heterogeneous enhancement ((**F**): corticomedullary phase; (**G**,**H**): nephrogenic phase).

DWI has three main applications for the assessment of renal masses. First, low-b EPI tracing can be used as a surrogate for FS T2W imaging, reducing the examination time [142]. Secondly, the long b-value of the EPI trace results in increased visibility of the renal lesion and thus an improvement in lesion detection [143]. Finally, the presence or absence of diffusion restriction can be a useful finding in the characterization of renal lesions. In this regard, several authors have reported encouraging results regarding the role of DWI in the characterization of solid renal masses, suggesting its usefulness especially in cases where the use of gadolinium is contraindicated or when enhancement is ambiguous. Indeed, several authors have demonstrated lower ADC values in malignant versus benign renal lesions, especially in RCC versus oncocytoma [144,145,146]. Kang et al. suggested a moderate accuracy of DWI for the prediction of malignancy and high-grade clear cell tumors [139]. A potential role of DWI has also been suggested in the differentiation of different RCC subtypes. In fact, significantly lower ADC values have been demonstrated in papillary (p)- and chromophobe (c)-RCCs than in cc-RCC (Figure 7) [147,148,149].

However, benign lesions could show restricted diffusion. Both typical and atypical AMLs can exhibit diffusion restriction, showing ADC values like those of p-RCC [150]. In addition, chronic hemorrhagic cysts may also show diffusion restriction due to internal viscosity and “black-out T2” effects [151], so low ADC values can also be found in pyelonephritis and abscesses [152].

### 6.4. Gadolinium-Enhanced Sequences

**Figure 8:**
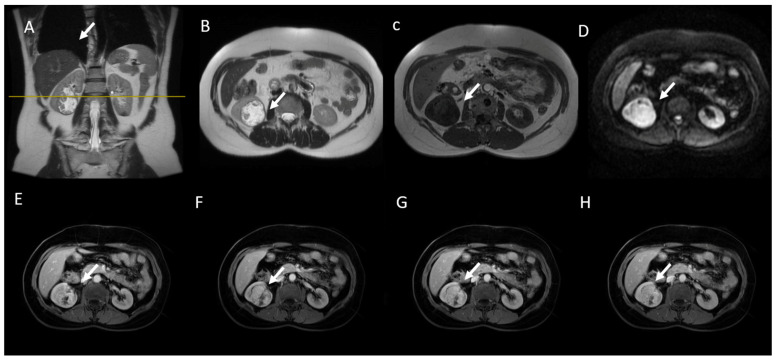
MRI assessment of RCC. (**A**) Coronal and (**B**) axial T2-weighted images show a heterogeneous hypointense renal mass (arrow). (**C**) Axial in-phase T1-weighted image shows a drop in signal intensity with restricted diffusion (**D**). Corticomedullary phase (**E**,**F**) and nephrographic phase (**G**,**H**) axial 3D fat-saturated GR T1-weighted images show mild enhancement.

The use of FS T1W GRE sequences, before and after gadolinium administration, is necessary to obtain a complete MR protocol [153,154]. The presence of enhancement after contrast administration is the key finding in the differentiation of cystic lesions from solid neoplasms. According to the Bosniak 2019 classification, enhancement is defined as an increased signal intensity greater than 15% of a renal mass in post-gadolinium T1 images compared to the pre-contrast images; both images should have been obtained with the same acquisition parameters [89,155,156]. A subtraction of T1 images allows for a better evaluation of subtle enhancement, presenting a sensitivity of 99% for solid renal tumors [153]. Furthermore, due to a higher contrast resolution, MRI has been shown to allow for a more adequate characterization of SRMs than CT by being able to overcome the limitation of pseudoenhancement (Figure 8) [102].

The acquisition of postcontrast images with the dynamic contrast enhancement (DCE) approach offer the possibility of obtaining quantitative parameters. Several authors showed that cc-RCCs have greater enhancement than the renal cortex, while p-RCCs increase less than the renal cortex [118]. In addition, it was seen that DCE can be useful for the differentiation of fat-poor AML from p-RCC, since p-RCC show a slower enhancement than AML [157]. On the other hand, one study showed that in using DCE-MRI, oncocytoma could be distinguished from p-RCC with high specificity due to delayed enhancement [123]. Finally, the utility of DCE has also been demonstrated in distinguishing tumor grade; a small series identified increased perfusion values in higher-grade tumors [158].

### 6.5. MRI in Bosniak Classification

The Bosniak 2019 classification also included MRI findings in the classification of cystic renal masses:

- Bosniak I cysts appear as well-defined masses with a smooth, thin wall (≤2 mm), homogeneous simple fluid with a signal intensity (SI) similar to that of cerebrospinal fluid (CSF) and without septa or calcifications. The wall may show contrast enhancement.
- Class II includes three types of cystic lesions, all of which are well-defined and have thin (≤2 mm) and smooth walls:
  1. The first type has lesions with thin (≤2 mm) and few (one to three) septa. The septa may have enhancement or calcifications of any type. Calcifications are less evident in MRI than in CT.
  2. The second type shows homogeneous and marked T2 hyperintensity (i.e., like that of cerebrospinal fluid) in the MRI without contrast.
  3. The third type exhibits homogeneous and marked hyperintensity on unenhanced, fat-saturated T1-weighted images (i.e., signal intensity ≥2.5 times more intense than adjacent renal cortical parenchyma) and typically includes a benign mass, hemorrhagic or proteinaceous [159,160].
- The IIF type is a non-enhancing and heterogeneously hyperintense lesion with no contrast in the T1W image. This type of lesion is important because sometimes RCCs, especially papillary subtypes, are hemorrhagic and may show mild or absent enhancement [161].

Finally, Classes III and IV in MRI include lesions with the same findings reported in the CT evaluation.

### 6.6. Imaging Tools and Renal Lesions: Advantages and Limits

The Bosnian classification [89] was originally developed to classify renal cysts based on CT findings, but MRI and CEUS can also be used, and the latter tools have greater sensitivity than CT for detecting renal microvasculature. Furthermore, the absence of ionizing radiation makes MRI and CEUS the preferred techniques for follow-ups.

Regarding solid lesions, CT and MRI are the recommended imaging modalities used to characterize them, since ultrasound is not sufficiently accurate. The accuracy of CT and MRI in characterizing renal lesions based on morphology and enhancement patterns is similar. Most guidelines recommend a preferential use of CT for the characterization of renal masses due to its greater availability, lower cost, better spatial resolution, and artefact-free quality images, and suggest the use of MRI for inconclusive and difficult cases [1,2]. However, the absence of radiation and the additional data provided by DWI sequences and DCE-MRI make MRI a more attractive and comprehensive technique, and therefore, depending on its availability, it can be considered the first diagnostic option. Furthermore, the choice will depend not only on the test initially performed, but also on the experience of each center, with different complementary techniques, possible contraindications, and other patient characteristics. CEUS can also be used in different scenarios [1] with the advantage of real-time evaluation, which allows for continuous evaluation in all phases, with the further advantage of the absence of radiation and the absence of nephrotoxicity in the means of ultrasound contrast.

### 6.7. Imaging Guided Percutaneous Biopsy

Thanks to the progress in techniques and the contemporary development of histological analyses, in recent years, imaging-guided percutaneous biopsy has acquired an increasingly key role in the characterization of SRMs, so much so that it is currently used routinely in some centers [162,163,164]. The advantages of histologic diagnosis are obvious and include the identification of surgical lesions versus those left undetermined on imaging; the identification of the specific tumor subtype and grade to better define prognosis and treatment; and the histologic confirmation of malignancy before the initiation of ablative treatments such as radiofrequency ablation and cryotherapy [165]. Current percutaneous imaging-guided biopsy techniques have demonstrated sensitivity values of 70–100% and specificity values of 100% [166]. However, reduced diagnostic performances have also been found for biopsies in SRMs. For example, Rybicki et al. reported higher sensitivity and negative predictive values in biopsies performed on 4–6 cm lesions than those performed on masses smaller than 3 cm, 97% and 89% versus 85% and 60%, respectively [167].

Improved techniques have also led to a significant decrease in post-procedural complications, including seeding, resulting in a currently good safety profile [168,169].

## 7. Radiomics

Radiomics is a field of medical research that uses programmable detection tools to extract objective information from standard images to be combined with clinical data to increase the diagnostic, prognostic, and predictive accuracy over standard vision interpretation [170,171,172,173,174]. Due to the large number of quantitative features in contemporary tumor imaging, such as the histogram (first-order statistics), texture (distribution of gray levels or second-order statistics), and shape, high-throughput data extracted from CT and MRI examinations are used to develop new radiomic markers for diagnostic, therapeutic, and prognostic purposes [175,176,177,178,179,180].

The use of radiomics in the evaluation of renal masses is an emerging and increasingly central field with diverse applications, such as characterizing renal masses, distinguishing subtypes of RCC, monitoring responses in target therapies, and prognosis in metastatic settings [181,182,183]. Another area of particular interest and development in radiomics relates to the improved characterization of SRMs; as mentioned above, although technological improvement has been achieved, conventional imaging still has some limitations in reliably differentiating between benign and malignant renal tumors [184]. Indeed, only a biopsy of the indeterminate renal mass currently provides an accurate diagnosis of the lesion, thus preventing any overtreatment [185]. However, there is still resistance to performing biopsies from the urologic community due to fears of needle tract infiltration, pathologic upstaging, and diagnostic uncertainty about oncocytic tumors [186,187].

Regarding the differentiation of benign from malignant renal tumors, several studies performed on CT and MRI images demonstrated better diagnostic performances using radiomics models compared to expert radiologists. Yap et al. performed a large retrospective study on CT images extracted from 735 patients (539 malignant and 196 benign masses), segmenting primary tumors and calculating 33 shape and 760 texture metrics per tumor [188]. They showed that the shape features alone reached an AUC between 0.64 and 0.68 on multiple classifiers, compared with 0.67–0.75 and 0.68–0.75 obtained from plot-only and combined models, respectively. Erdim et al. also performed a smaller retrospective study by extracting 271 texture features from the CT images of seventy-nine patients with 84 solid renal masses (63 malignant and 21 benign) and demonstrating an AUC of 0.915 for the differentiation between malignant and benign tumors [189]. Other authors have conducted retrospective studies to investigate the usefulness of radiomics models in the differentiation of benign versus malignant renal tumors using CT images, thus demonstrating better performances with machine learning than experienced radiologists in terms of sensitivity and specificity [190,191].

Regarding radiomics models applied to MRI images, Xi et al. conducted a large study on 1162 kidney lesions (655 malignant and 507 benign), showing better performances with the more optimized radiomics models than that of the average of expert radiologists with the evidence of a higher test accuracy (0.70 vs. 0.60, *p* = 0.053), sensitivity (0.92 vs. 0.80, *p* = 0.017), and specificity (0.41 vs. 0.35, *p* = 0.450) [191].

Concerning the differentiation between small fat-poor AML from RCC, several studies have demonstrated the discriminative efficiency of machine learning-based classification models performed on CT and MR images [192,193,194,195,196]. Yang et al. performed a retrospective study on 118 RCCs and 45 AMLs, extracting data from images at each stage of multiphasic CT and entering them into 224 classification models with multiple classifiers, resulting in 3360 discriminative models to be examined for higher-level features [197]. It emerged from this analysis that models achieving an AUC of 0.90 for differentiating low-fat AMLs from RCCs were those based on unenhanced CT alone or in association with nephrographic phase imaging. Shape and histogram features showed a greater discrimination ability than texture features. Feng et al. performed an analysis on a smaller series that included seventeen fat-poor AMLs and 41 RCCs using three-phase CT images to determine the best discriminatory classifiers [198]. Moreover, Razik et al. performed a retrospective study regarding the use of MRI-based texture analysis for differentiating low-fat AML and oncocytoma from RCC [30], thus reporting AUC values > 0.8 for 54 lesions (34 RCCs, 14 low-fat AMLs, and 6 oncocytomas) [199].

Also, relevant to the differentiation of RCC subtypes and oncocytoma, some authors demonstrated the utility of radiomics models based on data extracted from CT images. Coy et al. performed a retrospective study on data extracted from contrast CT images during the excretory phase of 179 renal lesions, including 128 cc-RCCs and 51 oncocytomas with a mean size of 3.8 cm (range 0.8–14.6 cm) and 3.9 cm (range 1.0–13.1 cm), respectively [200]. It was noted that radiomics models predicted oncocytoma with an accuracy of 74.4%, a sensitivity of 85.8%, and a positive predictive value (PPV) of 80.1%. Yu et al. performed another retrospective study on histogram features based on the CT images of 119 oncocytomas and other RCC subtypes, showing excellent AUC values of 0.93 (*p* < 0.0001) for differentiating cc-RCC from oncocytoma, 0.99 (*p* < 0.0001) for differentiating papillary RCC from oncocytoma, and 0.92 for differentiating oncocytoma from other subtypes [201]. Finally, another study demonstrated an AUC of 0.85 for CT-based quantitative features in differentiating chromophobe RCC (c-RCC) from oncocytoma in sixty-one histologically confirmed patients (44 c-RCCs, 17 oncocytomas) [202].

However, an important limitation of current studies for characterizing renal masses remains their heterogeneity in describing workflow characteristics, as highlighted by some systematic reviews [203,204]. Therefore, external and independent prospective validation studies and a diagnostic accuracy with reproducible and uniform radiomic features will be necessary to achieve the clinical application of radiomics [205,206,207,208,209,210,211,212,213,214,215,216,217,218,219,220,221,222,223,224,225,226,227,228,229,230,231,232,233,234,235,236,237,238,239,240,241,242].

## 8. Conclusions

The incidental findings of SRMs in patients undergoing imaging examinations to evaluate other conditions is a common event. Thereafter, management depends on the characterization of the SRMs via imaging, which allows in most cases to distinguish them as surgical or nonsurgical. This requires the correct knowledge of the advantages and limitations of the different imaging methods available and the patient’s clinical–anamnestic context. The adequate characterization of SRMs is already achieved via contrast-enhanced CT examination. Multiparametric MRI has been becoming, in recent years, the imaging method of choice in the characterization and management of SRMs, especially in cases of lesions indeterminate on CT. CEUS may also provide relevant information for the characterization of SRMs; however, its role remains controversial and further studies will be needed to clarify it.

Percutaneous biopsy presents a key role in characterizing lesions that remain indeterminate after imaging evaluation or before ablative treatment.

Radiomics is an emerging and promising field for the characterization of SRMs, having the potential for unlimited applications, thus being able to contribute to the realization of personalized medicine.

However, at present, radiomics is not yet ready to be used in clinical practice, and it will be necessary to wait and further share data algorithms, methodologies, and prospective validation studies.

## Data Availability

All data are reported in the manuscript and at link https://zenodo.org/records/10517723, (accessed on 16 January 2024).
